# A randomized controlled trial of the effectiveness of a pre-recruitment primer letter to increase participation in a study of colorectal screening and surveillance

**DOI:** 10.1186/1471-2288-14-44

**Published:** 2014-04-01

**Authors:** Christine Paul, Ryan Courtney, Rob Sanson-Fisher, Mariko Carey, David Hill, Jody Simmons, Shiho Rose

**Affiliations:** 1 Priority Research Centre for Health Behaviour (PRCHB), University of Newcastle, Callaghan, NSW, Australia; 2 Hunter Medical Research Institute (HMRI), New Lambton Heights, NSW, Australia; 3 Centre for Behavioural Research in Cancer, The Cancer Council Victoria, Carlton, VIC, Australia; 4 Professorial Fellow, University of Melbourne, Parkville, VIC, Australia; 5 Cancer Prevention Centre, The Cancer Council Victoria, Carlton, VIC, Australia

**Keywords:** Colorectal cancer, Patient recruitment, Population registers, Randomized controlled trials

## Abstract

**Background:**

Recruiting cancer patients is a barrier often encountered in research trials. However, very few randomized trials explore strategies to improve participation rates. The purpose of this study was to evaluate the effectiveness of a pre-recruitment primer letter to recruit persons diagnosed with colorectal cancer for a research trial.

**Methods:**

Potentially eligible participants were identified by the Victorian Cancer Registry. A total of 1,062 participants were randomized to receive either a mailed explanatory primer letter designed to encourage research participation, or no primer letter. Two weeks after the intervention, the Victorian Cancer Registry sought permission from patients to release their contact details to researchers. Those who agreed were contacted and invited to the study.

**Results:**

Pre-recruitment encouragement was not effective at increasing recruitment, with no significant differences demonstrated between experimental groups. Overall, 40% (n = 425) consented to participate, 25% (n = 243) refused and 35% (n = 394) did not respond.

**Conclusions:**

While this study demonstrated disappointing outcomes, pre-recruitment letters should not be ruled out as an approach altogether. Rather, future research should explore whether other factors to increase motivation, such as intensity and timing, are feasible and acceptable for contacting cancer patients.

**Trial registration:**

Australian and New Zealand Clinical Trials Registry, ACTRN12609000628246

## Background

### Methodological importance of attaining high response rates

Producing high quality, generalizable data from research trials is partly dependent on achieving high participation rates [1]. Response or participation rates are a direct determinant of whether the data accurately represent the group of interest [1]. Accordingly, guidelines for reporting trials (e.g. CONSORT [2]) and tools for evaluating the methodological quality of trials [3,4] emphasize the importance of high participation rates, with a high rate often considered to be 60% or more [5]. In the case of trials involving cancer patients, participation rates are often below 60%, regardless of whether the trial involves medical treatments [6], or supportive care [7]; or whether recruitment is via clinics [8] or cancer registries [9].

### Increasing participation of cancer patients in survey or cross-sectional research studies

Research regarding survey participation has identified incentives, personalization, reminders, and primer (pre-notification) letters as potentially effective strategies for enhancing response rates to research with varied populations [1,10]. The few studies trialing methods for increasing survey response rates specifically among cancer patients have produced mixed findings. A randomized controlled trial (RCT) of monetary incentives and survey length produced no significant differences in survey response rates for breast, prostate and colon cancer patients [11]. Modest effects were found for incentives on survey response rates for colorectal cancer (CRC) patients and their first degree relatives (FDRs) [12], while another RCT found no effect when comparing an enhanced letter to a standard invitation letter in hematological cancer patients (response rate = 37%) [9]. The effectiveness of mailed reminders has been demonstrated for head and neck cancer patients [13], with reminders at two and four weeks increasing survey response rates by 22% and 15% respectively to achieve an overall response rate of 79.6%.

### Testing the effectiveness of strategies to increase cancer patient participation in trials

Despite the importance of achieving high trial participation rates, very few RCTs focus on recruitment strategies relating to cancer patients. A systematic review [14] assessing interventions for patient participation in cancer treatment trials identified six studies, three of which were RCTs. None found an increase in trial recruitment. Strategies involved simplified documentation, a two-stage consent process, and additional trial information [14]. Two subsequent RCTs using audio-visual presentations found no effect among breast [6], colorectal, or lung cancer patients [15]. A review of 37 studies involving populations other than cancer patients found incentives, additional mailed contacts and interactive recruitment approaches were effective [16]. The larger pool of studies and positive findings among non-cancer patients suggest it is worthwhile to continue to develop and test such strategies for cancer-patient populations. Incentives, while effective for some groups, are often not appropriate for recruitment to treatment trials, while interactive options are not suitable for all settings. Therefore, print-based approaches are worthy of further exploration.

Population-based cancer registries provide centralized access to recruitment of cancer patients [17]. The use of a primer letter is an appropriate, low-cost strategy and may be beneficial where there is no personal contact, such as recruitment via cancer registries.

This study aimed to examine whether a primer letter compared to no primer letter, would improve research participation in CRC patients selected from a cancer registry. Given the importance of participant retention in trials, the study also explored whether receipt of the primer was related to completion of the first study follow-up at 12 months post-recruitment.

## Methods

### Sample and procedure

This study was conducted as part of the baseline phase of a larger RCT aiming to improve surveillance among those with CRC and improve appropriate screening among their FDRs [18]. The trial involved persons registered with the Victorian Cancer Registry (VCR) as being diagnosed with CRC. All diagnoses of cancer in Victoria are notified to the VCR. The primer letter study involved only the index cases.

Potentially eligible participants (18 years or older, a primary diagnosis of CRC, and within 3 months of diagnosis) were randomized by the VCR, to receive either a mailed primer letter (experimental group) or no primer letter (control group). Randomization was performed by using a randomized block design with the randomization sequence generated using Proc Plan in SAS. Participant eligibility criteria were based on the need of the larger RCT to provide intervention to patients soon after initial treatment. A process of rolling recruitment was used until assignment of groups was complete. Allocation was concealed to patients and researchers, with patient assignment revealed to researchers at conclusion of the trial. Randomization occurred at the point of diagnosis confirmation at the VCR. Simultaneously, a letter was sent to each patient’s treating clinician regarding whether the patient was well enough to participate in the main trial and had sufficient fluency in English to complete the study. The VCR adopts a passive approach to recruitment, therefore, if clinicians did not respond, the potential participant was considered eligible. Those considered ineligible by their clinician were excluded.

**Figure 1:**
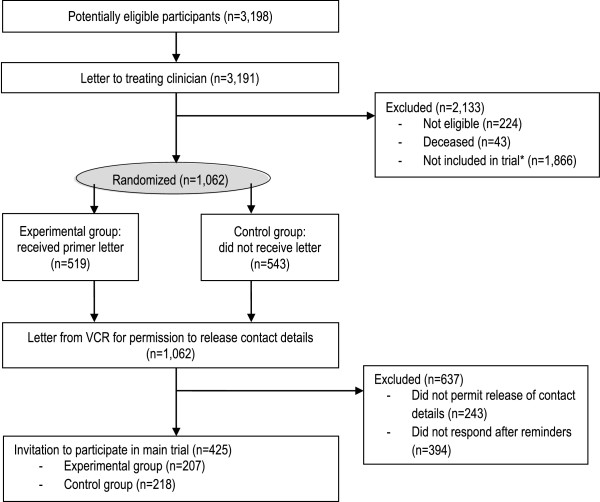
**Diagram of recruitment procedure.** *‘Not included in trial’ are those that were outside 3 months of diagnosis and those who were outside the trial period.

Consent into the study was a two stage process. Firstly, at approximately two weeks following the primer letter, the VCR contacted eligible patients by mail for permission to release their contact details to the researchers. Secondly, those who agreed to be contacted by researchers, were invited to participate in the study by mail, with two reminder letters sent to non-responders. Written consent to participate in the main trial and completion of the 12 month follow-up survey were used as the primer letter study outcome measures. Figure 1 describes the recruitment process.

Human research ethics approval for both the main trial and this study were obtained from The University of Newcastle and The Cancer Council Victoria, and registered with the Australian and New Zealand Clinical Trials Registry, registration number ACTRN12609000628246.

### The primer letter

The primer letter (appendix 1), designed to encourage research participation, was signed by the Director of the Cancer Council Victoria (CCV), highlighting: i) the importance of participation in research studies; ii) the role the VCR plays in assisting recruitment to research studies; and iii) the benefits to the individual and to the community of research participation. The letter followed behavioral principles for effective communication [19] including having a reading age of school year eight, simple sentence structure and repetition of main points. The primer letter required no response from participants and emphasized participants’ right to choose whether or not to be involved with research should they be invited to do so.

### Statistical methods

The primary outcome of the study was to detect differences in participation rates (i.e. consent for study invitation) between experimental groups. Patients were randomized until a sample size of approximately 1,000 was obtained to provide 80% power, with a 5% significance level, to detect a difference of 9%. Additional eligible patients on the registry were entered directly into the main trial. Consenting participants were compared between experimental groups using the chi-square test of independence.

## Results and discussion

Of the 1,062 eligible patients, 425 (40%) provided consent to make their details available to the researchers, 243 (25%) refused, and 394 (35%) did not respond to the mailed invitation. Persons more likely to consent to the trial were under 70 years and born in Australia. There was no difference in disease characteristics between the groups. As shown in Table 1, those receiving a primer letter were not significantly more likely to consent compared to persons not receiving a primer letter (χ^2^(2df) = 3.83, *P = 0.147*). Of those who consented, 296 (70%) completed the study baseline measure and 212 (50%) completed the 12 month follow-up measure. There was no significant difference between experimental groups in completion of baseline or follow-up measures (χ^2^(1df) < 1.6, *P > 0.21*).

**Table 1:** Response rates to receive invitation in main trial between those receiving versus not receiving a primer letter

	**Response type**			
	**Consentn (%)**	**Refusaln (%)**	**No responsen (%)**	**Total**
*Primer letter*	207 (40%)	131 (25%)	181 (35%)	519
*Control*	218 (40%)	112 (21%)	213 (39%)	543
**Total**	425	243	394	1,062

The study findings indicated that pre-recruitment encouragement was not effective in increasing recruitment to a subsequent trial relating to surveillance for people diagnosed with CRC. This finding is in accord with the few prior trials of recruitment-enhancing strategies for trials with cancer patients [6,15].

The finding is, however, surprising given the success of pre-notification strategies with non-cancer groups. Pre-notification strategies have two primary elements. The first relates to attention and preparation - raising the likelihood that an invitation to participate in a trial will be noticed or considered given it has been heralded on a prior occasion. The second element relates to raising motivation via an appeal to altruism. One potential explanation for the null finding is that these two potential effects are small and short-lived (if they occur at all) in the case of cancer patients. The invitation to participate may need to be very close to the primer event, or the primer may need to be intensive to exert an effect over and above the psychological distress often associated with the diagnosis and treatment of cancer. In this study, there was approximately two weeks between the primer letter and the invitation to participate in the trial.

Previous studies exploring barriers to participation in clinical cancer trials indicate patients prefer the freedom to explore medical treatments outside of trials [20], and may lack interest in trial participation [7]. Interest and motivation appear to be the more modifiable factors, and therefore, those most relevant to explore further. It is possible that in this case, the research participation letter was sent at a time when the study topic (improving disease surveillance among patients and screening in their FDRs) was not considered a priority to patients, as many may have been undergoing active treatment.

## Conclusions

While this study does not provide an endorsement of the pre-recruitment strategy, it is not sufficient to rule out the approach. Rather, future research should explore whether more intensive and timely approaches to increasing motivation are feasible and acceptable in the context of contacting cancer patients. Pre-recruitment primer letters may act as part of a persuasion strategy. The disappointing results of this study may justify the exploration of techniques such as testimonial references (e.g. ‘person like me’ endorsements) and compelling examples of the benefits in research participation, in the interests of enhancing the validity and generalizability of studies.

## Competing interests

The authors declare that they have no competing interests.

## Authors’ contributions

All of the authors have contributed to drafting and editing the paper; CP, RC, RSF, MC and DH contributed to development of the concept; RC was involved in data collection; CP and SR were involved in data analysis and interpretation. CP is guarantor for the paper. All authors read and approved the final manuscript.

## Pre-publication history

The pre-publication history for this paper can be accessed here:

http://www.biomedcentral.com/1471-2288/14/44/prepub
